# Diseases Admitted as in and Out-Patients under the Care of the Physicians of the Westminster Hospital, from the 20th of June to the 20th of July

**Published:** 1799-08

**Authors:** 


*Diseases admitted as In and Out-Patients under the care of the Physicians of the Westminster Hospital, from the 20th of June to the 20th of July.*

| | | | |
|---|---|---|---|
| Typhus - - - | 1 | Gastrodynia - - | 5 |
| Synochus - - | 7 | Hooping Cough - | 1 |
| Hepatitis - - | 1 | Hypochondriasis - | 3 |
| Measles - - - | 1 | Hysteria - - - | 2 |
| Amenorrhœa - - | 4 | Hydrocephalus - - | 1 |
| Asthenia - - - - | 2 | Impetigo - - | 2 |
| Asthma - - - | 1 | Itch - - - | 7 |
| Colic - - - - | 1 | Jaundice - - | 1 |
| Cough - - - - | 8 | Leucorrhœa - - | 1 |
| Diarrhœa - - | 1 | Phthisis - - | 3 |
| Dysuria - - | 1 | Quinzey - - | 1 |
| Dyspepsia - - - | 3 | Rheumatism - - | 12 |
| Dysentery - - | 2 | Struma - - - | 2 |
| Enterodynia - - | 3 | Urticaria - - | 1 |
| Erysipelas - - | 1 | Vomiting - | 1 |

As pectoral complaints, tending to phthisis pulmonalis, have been very frequent during the last and the preceeding month, trials have been made of the lichen Icelandicus. An ounce is boiled in a pint and a half of water down down to a pint; and two ounces of this decoction are taken three times a day. It does not appear to possess any antiphthisical virtues superior to other vegetable mucilages, but it sits pleasantly on the stomach, and is gently laxative, if not boiled too long.

